# Preparation of Microcapsules Coating and the Study of Their Bionic Anti-Fouling Performance

**DOI:** 10.3390/ma13071669

**Published:** 2020-04-03

**Authors:** Yu Li, Guoqing Wang, Zehui Guo, Peiqing Wang, Aimin Wang

**Affiliations:** 1 State Key Laboratory of Marine Resource Utilization in South China Sea, School of Materials science and Engineering, Hainan University, No. 58, Renmin Avenue, Haikou 570228, China; liyu@sidri.com (Y.L.); 13208916675@163.com (Z.G.); aimwang@163.com (A.W.); 2 Shanghai Investigation, Design & Research Institute Co., Ltd., Shanghai 200434, China; 3 Sichuan Sunvea New Materials Co., Ltd., Guangan 638500, China; wangpeiqing@sunvea.com

**Keywords:** microcapsules, silicone oil, capsaicin, anti-fouling coatings, micro-nano structure

## Abstract

With the increasing demands to better the marine environment, environmentally friendly anti-fouling coatings have attracted attention from society. Adding hydrolyzable microcapsules without toxin to paints is a very useful and safe method to get bionic anti-fouling coatings with a micro-nano surface structure. Based on this trend, a form of environment-friendly microcapsules were prepared through mini-emulsion polymerization. The target microcapsules had a poly(urea-formaldehyde) (PUF) shell and a mixed core of silicone oil and capsaicin. Additionally, the microcapsules were introduced into zinc acrylate resin to obtain bionic anti-fouling coatings with micro-nano morphology. The effects of polyvinyl alcohol (PVA) molecular weight, stirring rate, and temperature on the morphology of the microcapsules were studied by optical microscopy (OM), scanning electron microscopy (SEM) and transmission electron microscopy (TEM). It was found that spherical nanoparticles with smooth surfaces were obtained, and the mean diameter was approximately 1.38 μm when the molecular weight of PVA was 77 K, the stirring rate was 600 rpm and the temperature was 55 °C. Fourier-transform infrared spectra (FTIR) results showed that the silicone oil and capsaicin were successfully encapsulated, the core materials of the microcapsules reached 72.37% and the yield of microcapsules was 68.91% by the Soxhlet method. Furthermore, the hydrophobicity, corrosion resistance and anti-fouling performance of the coatings were evaluated by the water contact angle, electrochemical and real-sea tests. The results indicated that the anti-fouling coatings had excellent hydrophobicity and anti-fouling performance due to the micro-nano convex structure and the release of core materials. Encouragingly, the anti-fouling coatings show excellent and long-term anti-fouling performance, which is expected to be widely applied in marine anti-fouling coatings.

## 1. Introduction

Anti-fouling coatings usually rely on the release of toxins to achieve the effect of anti-fouling [1,2,3,4,5,6]. Conventional anti-fouling agents typically include toxic fungicides and heavy metals, which can cause different effects on the environment [7,8,9,10]. In recent years, the development of environmentally-friendly coatings to replace traditional coatings that contain tributyltin [11,12] and cuprous oxide [13,14] has attracted the interest of researchers. Therefore, it is necessary to discover an environmentally-friendly anti-fouling agent for use in anti-fouling paints, especially suitable for anti-corrosion in fish farming and offshore wind power.

Micro-nano anti-fouling coatings are currently a good direction for researchers exploring bionic anti-fouling paints [15,16,17,18]. This type of coating has a micro-nano structure that is generated at the surface by engraving or micro-electroforming methods to directly replicate biological surface morphologies [19,20,21]. Bers et al. [22] investigated five designed micro-textured surfaces and their effects on barnacle fouling and hydrodynamic drag; all micro-textured surfaces reduced recruitment, and the most efficient texture reduced recruitment by 98%. Schumacher et al. [23,24,25] researched the biological microstructure of shark skin, and the Sharklet^AFTM^ microstructure was developed using carved molds. This structure has been used to prevent the attachment of algae, barnacles and other fouling organisms, reducing the adhesion rate by approximately 85%. Compared to this method, chemical self-assembly is more flexible, cheaper and suitable for coating large areas, such as hulls. Li [26] developed a hybrid nanocoating composed of SBF and Ag@SBA to biomimetically simulate the lotus microstructure, and it exhibited good anti-biofouling effects. However, the use of silver increases the cost of these coatings. Liu [27] developed polyimide-copper anti-fouling coatings with unique capsular structures, which endow the polyimide coatings with interesting hydrophobicity and good anti-fouling performance, but the release of copper will affect the environment. Li et al. [28] synthesized a series of Cu_2_O micro-nano structures, and anti-fouling paints were prepared by using Cu_2_O with different morphologies as the marine anti-fouling agents. The results indicated that the prepared paints have good anti-fouling properties. Thus, for this problem, the strategy of constructing a new type of environmentally friendly bionic anti-fouling coating by the chemical self-assembly of an organic resin with microcapsules containing a non-toxic fungicide or mucus is expected to provide remarkable fouling control and enable the mimicry of biological epidermis structures and mucus, which would reduce the surface energy.

This study aimed to explore an effective method for the synthesis of microcapsules with sustained-release and anti-fouling properties. Additionally, the synthesized microcapsules were embedded in a zinc acrylate resin to determine the slow-release efficiency and the anti-fouling effect of the coating.

Therefore, to mimic the secretion of mucus on the surface of shark skin, an appropriate core material must be selected and then microencapsulated, and the resulting microcapsules can be utilized to control marine life by exploiting their sustained-release properties. Dimethyl silicone oil has a low surface tension, good hydrophobicity and good lubricity and is resistant to pollution, fouling and conglutination. Thus, dimethyl silicone oil specially with high degree of polymerization is non-toxic, which is often used in biomedical fields, such as antifoams drugs, asthma drop pills, antibacterial cleaning agents, etc., and also favored by people in the field of marine antifouling [29,30,31]. At the same time, to improve the anti-fouling performance, a non-toxic anti-fouling agent is encapsulated in the microcapsules with the silicone oil. Capsaicin, which is extracted from natural peppers and does not destroy the biological chain, can be used as a marine anti-fouling agent to kill plant spores and animal larvae attached to the outer surface of a ship and has be widely used in the field of anti-fouling materials [17,32]. Its irritating spicy flavor can also produce a series of physiological reactions and repellent effects on mammals, including humans and some pests. Capsaicin is a repellent that is extracted from natural peppers and does not destroy the biological chain. Compared to conventional anti-fouling agents, it does not have an impact on the environment.

The urea-formaldehyde resin was chosen as the shell material for the following two reasons. Firstly, poly(urea-formaldehyde) (PUF) has good mechanical properties and is cheap and easy to obtain. Secondly, PUF is a good candidate for the preparation of microspheres and microcapsules for the controlled release of an anti-fouling agent. Thus, silicone oil/capsaicin/PUF microcapsules can combine multiple performance properties to delay the release rate of the silicone oil and capsaicin, and improve the anti-fouling capacity to prolong the service life of the functional coating.

Microcapsules containing silicone oil and capsaicin as the core materials were prepared by a mini-emulsion method [33,34]. The effects of the molecular weight of PVA, agitation speed and temperature on the characteristics of the microcapsules were systematically studied. The microcapsules synthesized in the laboratory was mixed with zinc acrylate resin to evaluate their potential for producing anti-fouling coatings. At the same time, it is further compared with the coating with copper pyrithione to explore the coating with a better anti-fouling effect. The anti-fouling performance of the coating was assessed by measuring the water contact angle and the real-sea tests.

## 2. Experiments

### 2.1. Materials

Dimethyl silicone oil (*M*_w_ = 1.5 × 10^4^) and capsaicin were provided by Shanghai Sunvea Chemical Material Co., Ltd (Shanghai, China). Urea and formaldehyde (37 wt % aqueous solution), which were used as the monomers, were purchased from Shanghai West Long Chemical Co., Ltd (Shanghai, China). Resorcinol (99 wt %) and xylene were used as solvents and were obtained from Sigma Aldrich (Shanghai, China). Ammonium chloride (99.5 wt %) was supplied by Guangzhou Guanghua Technology Co., Ltd (Guangzhou, China). Sodium dodecylbenzene sulfonate (90 wt %, SDBS), gum arabic and polyvinyl alcohol (PVA) (Mw = 31 K, 77 K and 105 K), which was used as an emulsifier, were obtained from Sinopharm Group Chemical Reagent Co., Ltd (Shanghai, China). Hydrochloric acid (10 wt %) and sodium hydroxide (10 wt %) solutions were prepared in the laboratory and used to adjust the pH of the emulsion. 

### 2.2. Preparation of the Microcapsules (MOCs)

The microcapsules were prepared by a mini-emulsion method. Firstly, 0.1 g SDBS, 0.2 g PVA and 140 mL deionized water were placed into a 500 mL round-bottom flask at room temperature (22–25 °C). The 500 mL round-bottom flask was suspended in a temperature-controlled water bath on a programmable hotplate with an external temperature probe. The mini-emulsion was formed by slowly adding 5 g of a mixture of silicone oil and capsaicin (1:1) in xylene (the solvent) and emulsifying the mixture for 20–30 min at a rotor speed of 600 rpm. After the emulsion was stabilized, the pH of the solution was adjusted to 8.5 with sodium hydroxide and hydrochloric acid. Then, 2.5 g urea, 6.75 g of the 37 wt % aqueous formaldehyde solution, 0.28 g ammonium chloride and 0.28 g resorcinol were mixed into the solution, which was slowly heated to 55 °C at 1 °C/min and maintained for 4 h under stirring at 600 rpm. When the reaction was completed, the reaction solution was cooled, suction filtered and dried to obtain a free-flowing microcapsules powder.

### 2.3. Fabrication of the Coatings

#### 2.3.1. Electrochemical Impedance Spectroscopy (EIS) Testing Plate

Zinc acrylate resin as film former and iron oxide red as a pigment, adding 5%, 10% and 15% microcapsules, respectively, to prepare paints and one blank paint (without microcapsules), and then brush these paints to A3 carbon steel surface for EIS testing.

#### 2.3.2. Plate for Real-Sea Anti-Fouling Properties Testing

Zinc acrylate resin as film former and iron oxide red as a pigment, adding 5% of the microcapsules or copper pyrithione, respectively, to prepare two paints and one blank paint (without microcapsules or copper pyrithione), and then spray these paints to Q235 steel plate coated by 100 μm epoxy anti-corrosion coating to test the anti-fouling properties.

### 2.4. Characterization

#### 2.4.1. Optical and Scanning Electron Microscopy

One to two droplets of the prepared microcapsules emulsion were uniformly smeared on a slide, which was then placed on an optical microscope and observed under 100× magnification.

#### 2.4.2. Scanning Electron Microscopy (SEM) and Transmission Electron Microscopy (TEM)

The microcapsules were vacuum dried, and then the dried microcapsules powder was attached to the sample stage by conductive adhesion with gold sputtering at 50 MA for 20 s. The morphology of the microcapsules was observed using a Quanta 400 FEG scanning electron microscope at 15 kV (FEI, Hillsboro, OR, America). The microstructures of the samples were determined by TEM (Hitachi H-800; JOEL, Tokyo, Japan) at an accelerating voltage of 100 kV. The specimen was dispersed in water, and some pieces were collected on carbon-coated 300-mesh copper grids for TEM observation.

#### 2.4.3. Fourier Transform Infrared (FTIR) Analysis

Fourier transform infrared spectra of the prepared samples were acquired using a Nicolet 6700 IR spectrophotometer (Bruker, Bremen, Germany) in the spectral range of 400–4000 cm^−1^ with a 4 cm^−1^ resolution for 18 scans. FTIR was used to characterize the valence bond structure, and the FTIR spectra of silicone oil, the pure UF resin (prepared in the laboratory), capsaicin and the microcapsules were analyzed to confirm that silicone oil and capsaicin were successfully encapsulated in the microcapsules.

#### 2.4.4. Soxhlet Extraction for the Core Material

The mass fraction of the core materials was determined by acetone extraction method, the ground microcapsules were extracted by a Soxhlet extractor (Anhui Weisi Experimental Equipment Co., Ltd, Anhui, China) for 48 h so as to remove the core materials. 

#### 2.4.5. Water Contact Angle Measurements

The water contact angle of the microcapsules coating was measured by a contact angle analyzer (JCY-3, Suzhou Qi Le Electronic Technology Co., Ltd., Suzhou, China) using a drop of deionized water. 

#### 2.4.6. Electrochemical Tests

The corrosion resistance of the coatings were examined using a Solartron Modulab system (CVB120, ZAHNER; Bio-Logic, Seyssinet-Pariset, France) under simulated marine conditions. All the tests were performed at room temperature in seawater. Before the electrochemical tests, the samples were immersed in seawater for 30 min. Using a conventional three-electrode cell, the prepared coatings were used as the working electrode with an area of 1 cm^2^ coated on A3 carbon steel surface. The reference electrode was a saturated calomel electrode, and the counter electrode was a platinum electrode. Potentiodynamic polarization curves of the coatings were obtained in the potential range from −1000 mV to 700 mV versus EOCP (Electrochemical open circuit potential) at a scan rate of 0.5 mV/s. EIS measurements were performed at an applied AC signal of 20 mV in the frequency range of 10^−2^ Hz to 10^5^ Hz. 

#### 2.4.7. Real-Sea Tests of the Coatings

Real-sea tests were conducted in the sea marine station of Haikou Bay of Hainan province in China. The investigations were performed in winter and spring, according to Chinese standard GB/T 5370-2007.

## 3. Results and Discussion

### 3.1. Formation Mechanism

**Figure 1:**
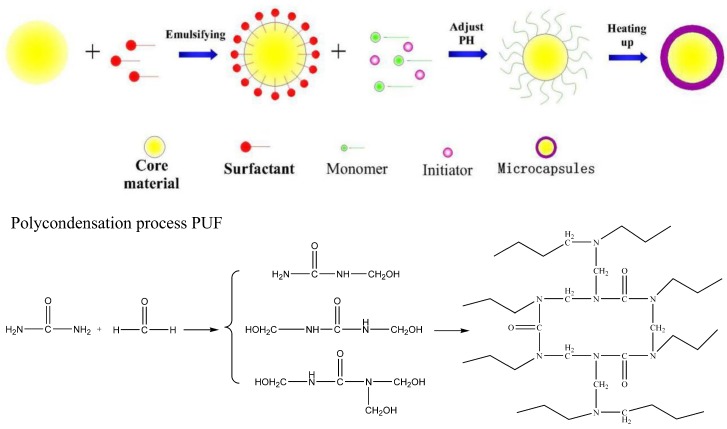
Mechanism of urea-formaldehyde microcapsule formation.

The scheme of the synthesis procedure for the microcapsules is shown in Figure 1. The emulsifier overcomes the cohesive energy of the oil phase and the surface energy for droplet formation, and the small oil droplets are sufficiently dispersed in the aqueous phase to form a stable emulsion under mechanical stirring. Subsequently, the monomers urea and formaldehyde are added to the aqueous phase and undergo a condensation reaction to form a linear pre-polymer on the surfaces of the oil droplets. The polymer is neither soluble in water nor soluble in the mixture of the oil phase and monomer. Due to the interfacial energy, the linear polymer can only be precipitated on the surface of an oil droplet, and the microcapsules are finally formed by curing and cross-linking. The linear pre-polymers are obtained under acidic and alkaline conditions. Under alkaline conditions, a series of reactions result in the formation of mono, dimethylol and trimethylol urea (Figure 1), followed by the condensation of methylol urea and the amino groups at high temperatures. After hardening, the oil droplets form a three-dimensional network of PUF resin. 

**Figure 2:**
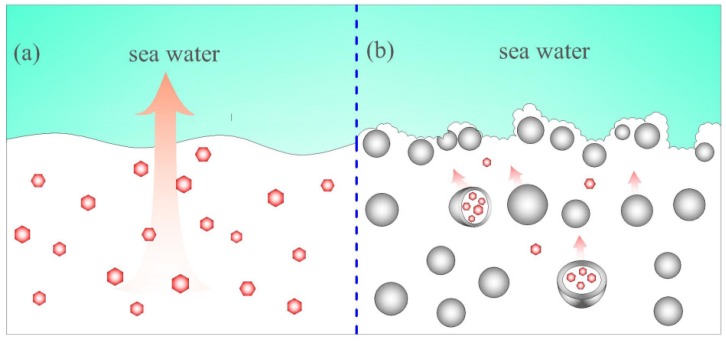
Dispersion of the anti-fouling agent in the coating by (**a**) a traditional dispersion method and (**b**) immobilization due to encapsulation.

The release rate of the anti-fouling agents from the coating is determined by their slow release from the microcapsules to the surrounding coating matrix, and the encapsulated biocide is protected from degradation [15]. As shown in Figure 2a, the anti-fouling agents that are molecularly dispersed in the coating are subject to fast release. Microencapsulation can solve the problem of premature release in coatings (Figure 2b).

### 3.2. Characterization of the Microcapsules (MOCs)

#### 3.2.1. Morphology of the MOCs

**Figure 3:**
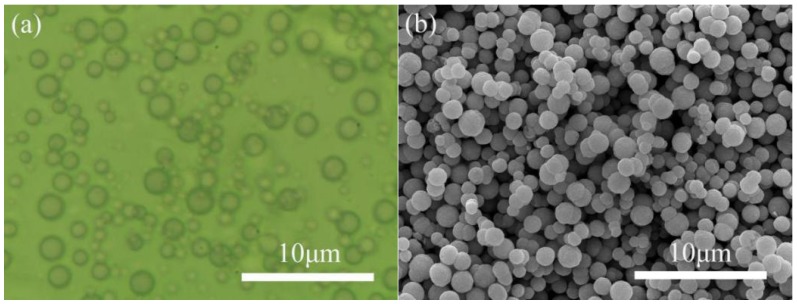
Morphology of the synthesized microcapsules: (**a**) optical microscopy and (**b**) SEM images.

**Figure 4:**
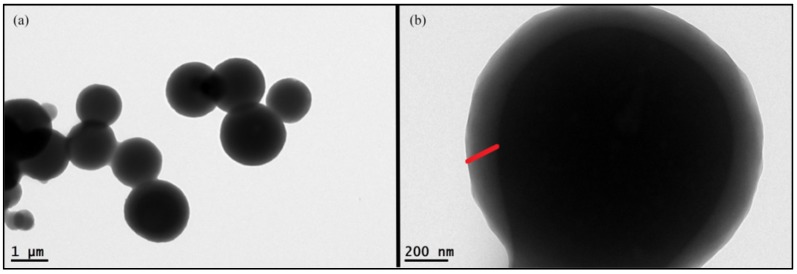
TEM images of the synthesized microcapsules. (**a**) ×8000 times; (**b**) ×40,000 times.

The morphology and microstructure of the microcapsules observed by SEM and TEM are shown in Figure 3 and Figure 4. The TEM images (Figure 4a,b) show that the particles have a well-defined core-shell structure with a shell thickness of approximately 170 nm. In the preparation process, the mixed solution of capsaicin and silicone oil is homogeneous, suggesting that capsaicin and silicone oil are successfully encapsulated in the PUF shell.

#### 3.2.2. Fourier Transform Infrared (FTIR) Spectroscopy

To confirm the successful synthesis of the microcapsules, FTIR is used to characterize the valence bond structure of PUF, silicone oil, capsaicin and the prepared microcapsules (MOCs).

The spectrum of silicone oil exhibits characteristic peaks due to the Si-O-Si stretching vibrations at 1126–1000 cm^−1^. A sharp peak appears at 1257 cm^−1^ and is attributed to the absorption of the terminal Si-CH_3_ at the end of silicone oil. The stretching vibration of C-H is observed at 2962 cm^−1^. Other sharp peaks are also observed at 840–670 cm^−1^ due to the stretching vibrations of Si-C.

**Figure 5:**
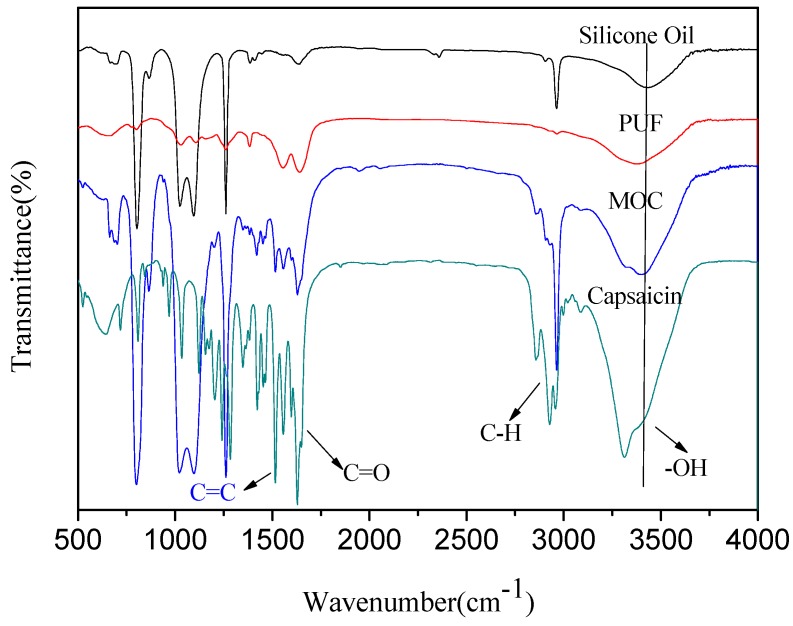
FTIR spectra of the pure poly(urea-formaldehyde) (PUF), the pure silicone oil, capsaicin and the synthesized MOCs.

The FTIR spectrum of the pure PUF is shown in Figure 5. The spectrum has a broad, strong absorption peak at 3380 cm^−1^. The peak corresponds to the stretching vibrations of N-H (3100–3500 cm^−1^) and O-H, the latter of which is due to absorbed water. A relatively obvious absorption peak appears at 1556–1642 cm^−1^ and corresponds to the C=O (-CONH_2_) stretching vibration of the linear polymer and the stretching vibration peaks of the secondary linearly polymerized -NH-CO- unit, indicating that urea and formaldehyde form a linear polymer. The other sharp peaks in the FTIR spectrum might be due to the cross-linking of the linear polymer. For example, the band at 1030 cm^−1^ corresponds to the ether bond in -CH_2_-O-CH_2_- formed by the hydroxyl and methyl groups during the cross-linking process, ensuring that the -NH bond is formed on both sides of the polymer.

**Scheme 1:**
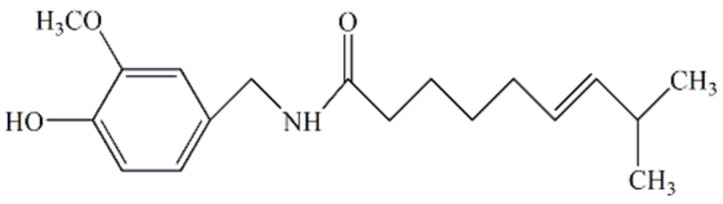
Chemical formula of capsaicin.

The typical absorption peaks of capsaicin, such as the N-H/O-H stretching vibrations at 3350 cm^−1^, the stretching vibration of the C-H bond at 2928 cm^−1^, the C=O stretching vibration at 1629 cm^−1^, the C=C stretching vibration at 1516.16 cm^−1^ and the -C-O-C- bond at 1034 cm^−1^, are observed in its FTIR spectrum. Chemical formula of capsaicin is shown in Scheme 1.

The FTIR spectrum of the microcapsules not only contains the N-H stretching vibration of PUF at 3409 cm^−1^ and the C=O absorption peak at 1641 cm^−1^ but also exhibits an Si-O-Si absorption peak at approximately 1025–1095 cm^−1^ and other characteristic peaks of silicone oil and capsaicin. This spectrum exhibits the characteristic peaks of silicone oil, capsaicin and the pure PUF. Therefore, the FTIR analysis demonstrates that silicone oil and capsaicin are successfully encapsulated in a poly(urea-formaldehyde) resin shell.

#### 3.2.3. The Yield and the Content of Core Material for Microcapsule

The yield of microcapsules (η) can be calculated by Equation (1) as follows. The core content (*M_core_*) was calculated by the following Equation (2):

(1) $$\mathrm{Yield}(\%)=\frac{M_{1}}{M_{m}+M_{s}+M_{c}+M_{\mathrm{SDBS}}^{}+M_{\mathrm{PVA}}+M_{a}+M_{r}}\times 100\%$$

(2) $$M_{core}(\%)=\frac{M_{1}-M_{core}}{M_{1}}\times 100\%$$

where *M*_1_ is the mass of microcapsules; *M_m_* is the mass of shell monomer, including urea, formaldehyde and ammonium chloride; *M_s_*, *M_c_*, *M*_SDBS_, *M*_PVA_, *M_a_* and *M_r_* are the mass of silicone oil, capsaicin, SDBS, PVA, ammonium chloride and resorcinol, respectively. The data is shown in Table 1.

Thus, the core materials of the microcapsules reach 72.37% and the yield of microcapsules is 68.91%.

#### 3.2.4. Effect of the Molecular Weight of the PVA Stabilizer

The molecular weight of the emulsifier PVA has an important effect on the stability of the emulsion in the reaction system. Large PVA molecules increase the viscosity of the continuous phase to form a steric barrier, which can effectively prevent the oil droplets from coalescing and fragmenting upon collision [27]. To explore the effect of the PVA molecular weight on the microcapsules, PVA samples with different molecular weights (31 K, 77 K, and 105 K) are selected. 

**Figure 6:**
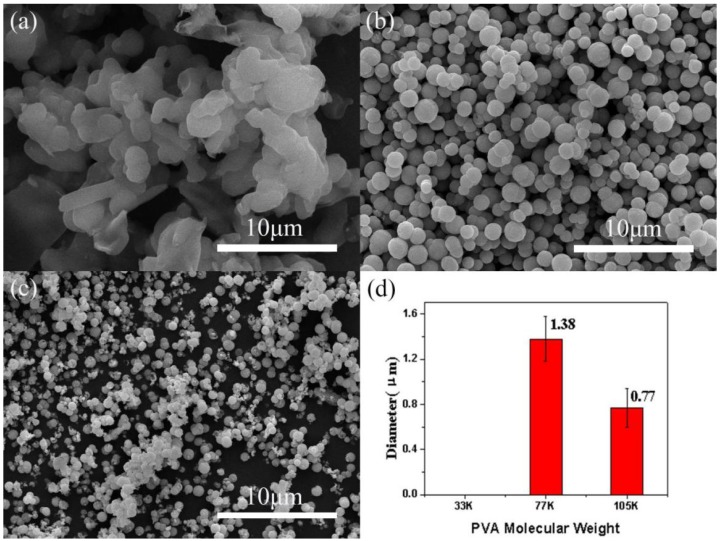
SEM images of the microcapsules (MOCs) synthesized using polyvinyl alcohol (PVA) with a molecular weight of (**a**) 31 K, (**b**) 77 K, and (**c**) 105 K; (**d**) mean diameters obtained using PVA with different molecular weights.

The effect of the PVA molecular weight on the surface morphology of the microcapsules is analyzed by SEM. As shown in Figure 6, when 31 K Mw PVA is used as the emulsifier, micron-sized agglomerates with an irregular morphology are obtained, indicating that 31 K Mw PVA cannot create a stable oil-in-water emulsion due to its low viscosity. When the molecular weight of PVA is increased to 77 K, spherical nanoparticles with smooth surfaces are obtained, and their mean diameter is 1.38 μm. However, for the microcapsules synthesized with 105 K Mw PVA, the mean diameter decreases to 0.77 μm, and some broken microcapsules are also observed, as shown in Figure 6c. This result might be explained by the increase in the viscosity of the continuous phase with increasing molecular weight, increasing in the drag force generated by the surface of the dispersed phase (oil drops) and thus a decrease in the mean diameter of the microcapsules.

#### 3.2.5. Effect of the Stirring Rate

**Figure 7:**
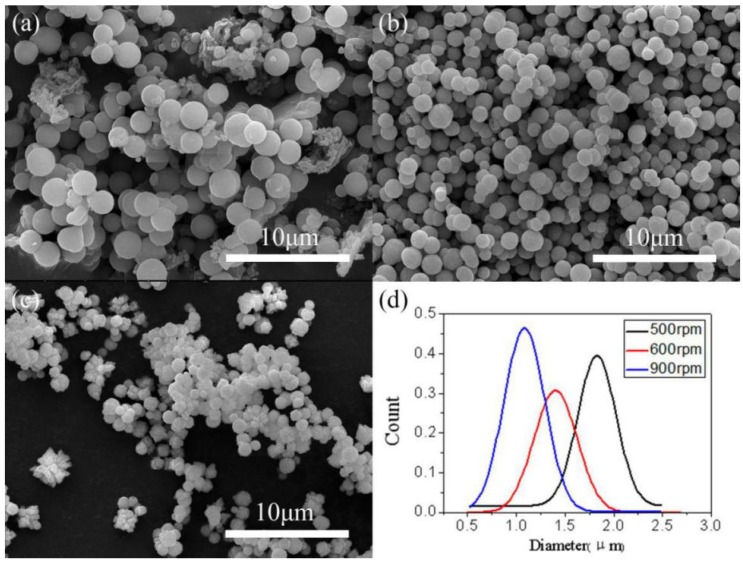
SEM images of the MOCs synthesized at (**a**) 500; (**b**) 600; and (**c**) 900 rpm; (**d**) fitted size distribution curves.

The stirring rate has a considerable effect on the particle size of the microcapsules, which can be regulated by adjusting the stirring rate. The agitation speed exerts a shear force that reduces the surface tension and changes the viscosity of the reaction system during the formation of the oil-in-water emulsion. The size and morphology of the microcapsules can be varied by changing the stirring rate during the preparation process. As shown in Figure 7, the particle size of the microcapsules decreases and the size distribution becomes narrower with increasing stirring rate. However, an extremely high stirring rate can lead to collisions between the droplets and the subsequent agglomeration of the microcapsules, thus lowering the yield [35]. The effect of the agitation speed on the particle size distribution is determined by characterizing more than 300 microcapsules fabricated at different agitation speeds. As the agitation speed is increased from 500 rpm to 900 rpm, the mean particle size decreases from 1.78 to 1.09 μm. This trend is observed in the fitted size distribution curves shown in Figure 7d.

#### 3.2.6. Effect of Temperature

**Figure 8:**
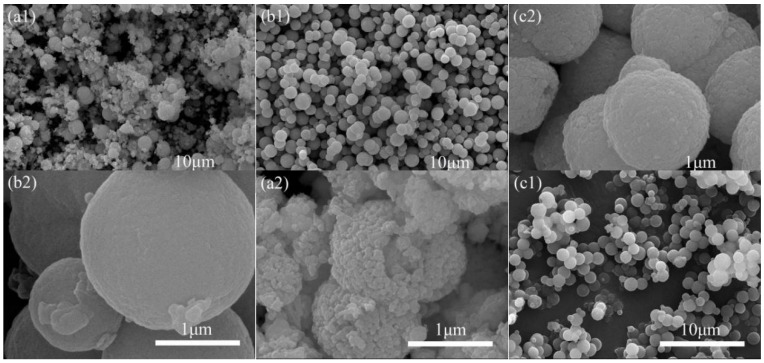
SEM images of the MOCs synthesized at (**a1**,**a2**) 50 °C; (**b1**,**b2**) 55 °C; and (**c1**,**c2**) 65 °C.

The effect of temperature on the properties of the microcapsules is investigated by observing the morphology of the microcapsules at 50, 55 and 65 °C. As shown in Figure 8a1 and a2, most of the microcapsules synthesized at 50 °C are ruptured. The image also reveals a large number of powdered impurities, which might be PUF that fails to cover the surface of the mixture (silicone oil and capsaicin) during the reaction at 50 °C. When the reaction temperature is increased to 55 °C, the microcapsules have an intact spherical shape with a smooth surface. For the reaction at 65 °C, the shell material of the microcapsules exhibits higher roughness compared to that obtained at 55 °C. The results can be explained by the kinetics of the urea-formaldehyde reaction [36]. In an acid medium, the rate of the reaction between urea and formaldehyde increases significantly with increasing temperature [26]. Therefore, as the temperature increases, the reaction of urea and formaldehyde to form PUF is accelerated, leading to the excessively fast deposition of resin oligomers on the surfaces of the oil droplets and the agglomeration of the formed microcapsules. Thus, performing the synthesis reaction at a higher reaction temperature results in a higher shell strength and surface roughness.

### 3.3. The Anti-Fouling Performance of the Microcapsule’s Coatings

**Figure 9:**
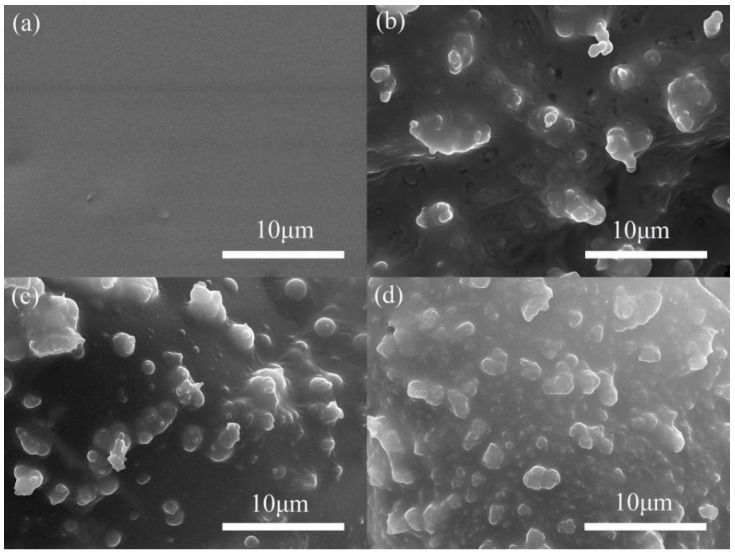
SEM images of the coatings (**a**) without microcapsules and with (**b**) 5%; (**c**) 10%; and (**d**) 15% microcapsules.

The SEM images of the coatings are shown in Figure 9. The microcapsules induce the formation of protrusions of approximately 2–5 μm in diameter on the surface of the coating. These surface features resemble the micro-nano structures on the surface of a lotus leaf [37].

#### 3.3.1. Contact Angles and Slow-Release Effect of the Microcapsules Coating

**Figure 10:**
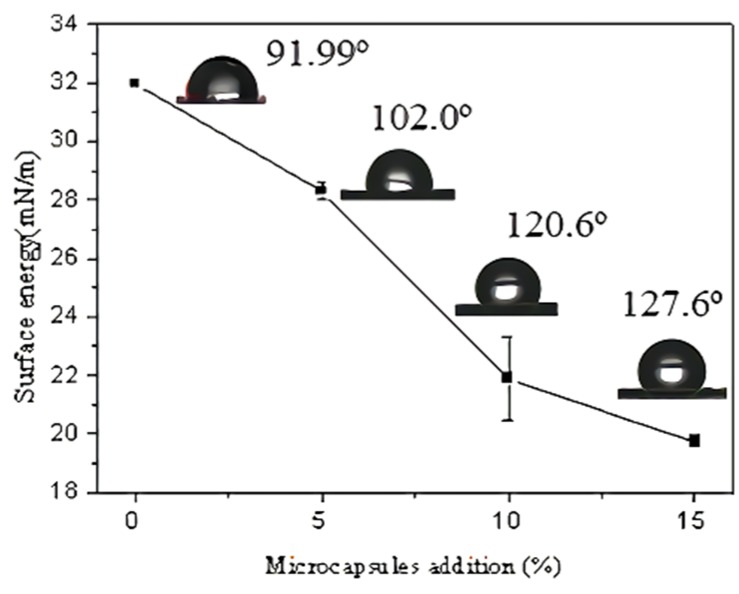
Water contact angles and surface energies of the coatings.

The data generated by the contact angle analyzer is reported in Figure 10. A closer look at the data indicates that the contact angles of the coatings are greater than 90° and that the surface energies are less than 30 mN/m, indicating that marine creatures should have difficulty attaching to them. The angle increases from ~91.99° for the pure resin coating to ~127.6° for the coating with 15 wt % microcapsules. The reduced surface energy is generally explained by the hydrophobicity due to the embedding of PUF microcapsules [36]. 

To further examine the slow-release effect of microcapsules in anti-fouling coatings, the slow-release properties of microcapsules by characterizing the change of water contact angles and the slow-release efficiency of capsaicin encapsuled in the microcapsules, which were shown in Supplementary Material Figure S1 and Figure S2, respectively. The core materials encapsulated has well slow-releasing property, thereby promoting coatings embedding microcapsules longer-lasting anti-fouling performance.

#### 3.3.2. Electrochemical Tests (EIS)

**Figure 11:**
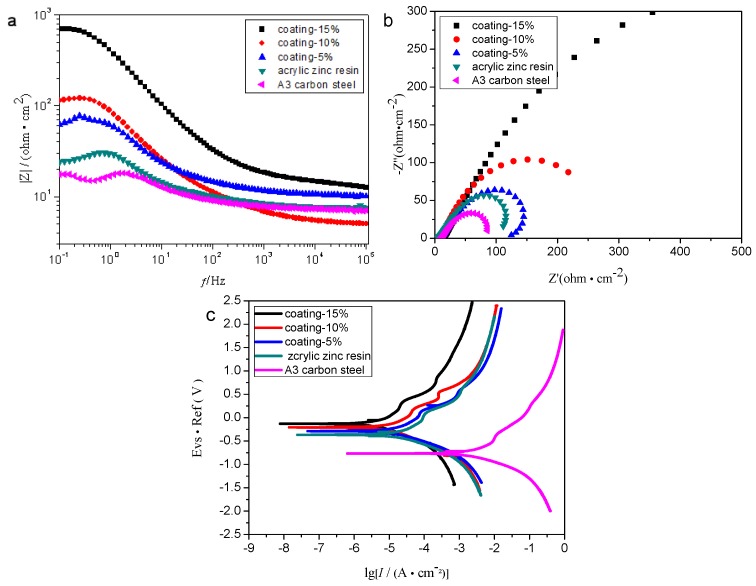
Electrochemical performance of the coatings: (**a**) EIS Bode plots; (**b**) EIS Nyquist plots, and (**c**) potentiodynamic polarization curves of the coatings in seawater.

Electrochemical tests are performed to further measure the corrosion resistance of the coatings since the corrosion resistance must also be evaluated for marine applications [38,39]. Nyquist and Bode plots and potentiodynamic polarization curves of the coatings in seawater are presented in Figure 11. The impedance spectra of the specimen are presented as Bode plots of EIS (Figure 11a). The results show that the coating with 15% microcapsules has a higher maximum impedance modulus |Z|. The Nyquist plots of the samples are shown in Figure 11b. The coating with 15% microcapsules exhibits the highest corrosion resistance, whereas the A3 carbon steel has the lowest corrosion resistance, demonstrating the higher corrosion resistance of the coatings.

The potentiodynamic polarization curves of the coatings are shown in Figure 11c. The curve parameters, such as the corrosion potential and corrosion current density, for the A3 carbon steel, zinc acrylate resin, and 5%, 10%, and 15% microcapsules coatings in seawater are listed in Table 2. The corrosion current density of the coating with 15% microcapsules is 1.903 × 10^−7^ A/cm^2^, which is lower than those of the other samples. The potentiodynamic polarization curves show that the coating with 15% microcapsules has a higher corrosion potential. The result reveals the good corrosion resistance of the coating with microcapsules.

The corrosion protection efficiency (PE) of the coating was calculated using the Equation (3) [40]:

(3) $$PE\left(\%\right)=\frac{Icorr,bare-Icorr,coated}{Icorr,bare}\times 100\%$$

where *Icorr, bare* and *Icorr, coated* are the corrosion current densities of the bare A3 carbon steel and the coating, respectively. The corrosion protection efficiency (*PE*) of the coating with 15% microcapsules is 82%.

#### 3.3.3. Real-Sea Tests of the Coatings

To investigate the long-term anti-fouling performance of the coatings, real-sea tests was conducted. Table 3 shows the real-sea results for the coatings in the Haikou Bay. After one month, the blank panel is already coated biofouling, and after four months, the biofouling gets worse. In contrast, as for the coatings with 5% microcapsules, marine organism are barely visible after one month, and there is only a few aquatic plants are attached after four months. At the same time, compared to the coating with 5% copper pyrithione, the coating with the 5% microcapsules also shows better and longer-term anti-fouling performance, so the heavy metal copper coating can be replaced by safe and nontoxic microcapsules coating. There may be two reasons why the microcapsule coating shows such an excellent anti-fouling effect: firstly, the surface of the microcapsules coating exhibits a micro-nano structure that biomimetically simulated the surface of a lotus leaf [41,42,43]; and secondly, the microcapsules could act as controlled-release non-toxic antifoulants and also secrete mucus to imitate the surface of shark skin, which significantly improves the hydrophobicity and anti-fouling performance of the coatings. These results indicate that the micro-nano convex structure and the slow-release effect result in a coating with excellent hydrophobicity and anti-fouling performance.

## 4. Conclusions

In this work, PUF microcapsules loaded silicone oil and capsaicin were successfully synthesized via a mini-emulsion polymerization, moreover, the microcapsules were mixed with zinc acrylate resin and iron oxide red to prepare bionic anti-fouling coatings. The morphology of microcapsules was characterized by OM, SEM and TEM. The results showed that the spherical nanoparticles with smooth surfaces were obtained, and the mean diameter was approximately 1.38 μm when the molecular weight of PVA was 77 K, the stirring rate was 600 rpm and the temperature was 55 °C. The FTIR spectra showed that silicone oil and capsaicin were successfully encapsulated in PUF, the core materials of the microcapsules reached 72.37% and the yield of microcapsules was 68.91% by the Soxhlet method. At the same time, the hydrophobicity, corrosion resistance and anti-fouling performance of the coatings were assessed by the water contact angle, electrochemical and real-sea tests. The results indicated that the anti-fouling coatings had excellent hydrophobicity and anti-fouling performance due to the micro-nano convex structure and the release of core materials. Furthermore, compared to the coating containing copper pyrithione, the target anti-fouling coating with 5% microcapsules showed a better and longer-term anti-fouling effect. In general, this study provides a novel anti-fouling composites, and their effects on anti-fouling in marine anti-fouling paints will be further investigated in our future study.

## Figures and Tables

**Table 1:** Synthesis conditions and main index of the microcapsules.

W_s_ (g)	W_c_ (g)	W_SDBS_ (g)	W_PVA_ (g)	W_a_ (g)	W_r_ (g)	Yield (%)	Core Fraction (%)
2.5	2.5	0.1	0.2	0.28	0.28	68.91	72.37

**Table 2:** Potentiodynamic polarization curve parameters for the A3 carbon steel, zinc acrylate resin, and 5%, 10%, and 15% microcapsule coatings in seawater.

Sample	Corrosion Potential (E_corr_) (V/SCE)	Corrosion Current Density (I_corr_) (A/cm^2^)
A3 carbon steel	−0.770	8.612 × 10^−5^
zinc acrylate resin	−0.371	1.084 × 10^−6^
5% microcapsule coating	−0.283	1.380 × 10^−6^
10% microcapsule coating	−0.194	6.589 × 10^−7^
15% microcapsule coating	−0.129	1.903 × 10^−7^

**Table 3:** Photos of the real-sea tests.

Paint	Immersion Time			
	0 Day	One Month	Two Months	Four Months
blank	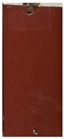	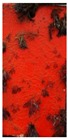	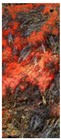	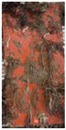
5% microcapsules	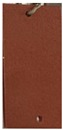	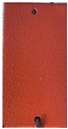	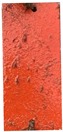	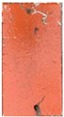
5% Copper pyrithione	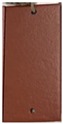	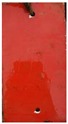	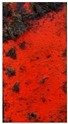	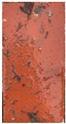

## Supplementary Materials

The following are available online at https://www.mdpi.com/1996-1944/13/7/1669/s1, Figure S1: Water contact angle of (a) the coatings embedding the target microcapsules and (b) the coatings filling silicone oil; Figure S2: The slow-release efficiency of capsaicin.
