# Supplementary material for: Preparation of Microcapsules Coating and the Study of Their Bionic Anti-Fouling Performance
Source: Materials (Basel). 2020 Apr 3;13(7):1669. doi: 10.3390/ma13071669 (PMC7178335; doi:10.3390/ma13071669)
Supplement: Supplementary file 1 [file materials-13-01669-s001.pdf]

materials MDPI

Supplementary Materials

1. Contact Angles under Cyclic Corrosion

To further examine the slow-release effect of microcapsules in antifouling coatings, the microcapsules were embedded into the resin to test the slow-release properties of microcapsules by characterizing the change of water contact angles. The coatings filling silicone oil and the coatings embedding the target microcapsules were used for comparison. The surface of coatings was eroded with tap water under the fixed flow velocity with 2 h as one corroding cycle. The water contact angles were measured after one corroding cycle following with one drying step which the drying conditions were cured at 80 °C for 3–5 min.

The resultant curves were present in Figure S1, which was shown the similar contact angles of two coatings at the beginning. With the increasing of corroding time, the contact angle of the coatings embedding the target microcapsules showed little changes. However, the water contact angle of the coatings filling silicone oil had a decreasing trend, and the only angle of 86.47° was obtained at the 16th cycle times. These comparative data distinctly demonstrate the well slow-releasing property of microcapsules and suggest that microencapsulation of silicone oil has a significant effect on marine antifouling paints with longer service life.

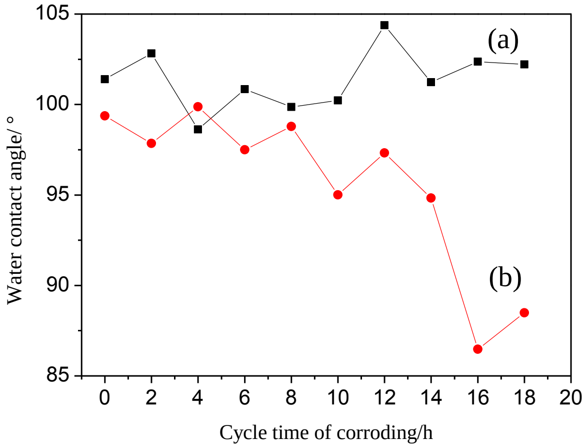

Figure S1. Water contact angle of (**a**) the coatings embedding the target microcapsules and (**b**) the coatings filling silicone oil.

2. The Slow-Release Efficiency of Capsaicin Encapsuled in the Microcapsules

2.1. Standard Curve of Capsaicin

Capsaicin standard curve: 10 μg/mL, 20 μg/mL, 30 μg/mL, 40 μg/mL, 50 μg/mL, 60 μg/mL, 70 μg/mL, 80 μg/mL capsaicin methanol solution was prepared accurately, the absorbance value of the solution was measured by UV-visible spectrophotometer at the wavelength of 280 nm, and then the concentration-absorbance curve was drawn. After linear fitting, the standard curve of capsaicin was $y = 0.00951x + 0.02626$, $R = 0.99755$. Where, y is the absorbance value and x is the concentration of capsaicin (μg/mL).

2.2. Slow-Releasing Property of Capsaicin

The 15% coated coating was placed in seawater, and was taken out at different intervals (d). Then, the solution was shaken and centrifuged, the supernatant was taken, and the absorbance of the solution was measured with a UV-visible spectrophotometer at a wavelength of 280 nm. The slow-release effect of capsaicin in the antifouling coating was determined according to the capsaicin standard curve. The sustained release efficiency of microcapsules was shown in Figure S2.

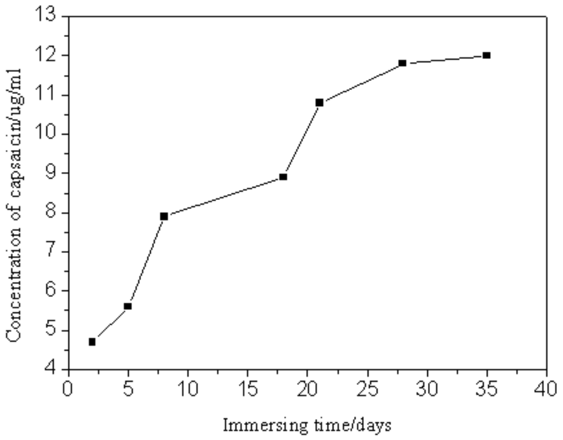

Figure S2. The slow-release efficiency of capsaicin.

© 2020 by the authors. Submitted for possible open access publication under the terms and conditions of the Creative Commons Attribution (CC BY) license (http://creativecommons.org/licenses/by/4.0/).
